# Factors affecting and the strategies to enhance emotional regulation among adolescents in South Asian countries: a systematic review

**DOI:** 10.1186/s12889-025-24793-8

**Published:** 2025-10-14

**Authors:** Preejana Sharma, Linu Sara George, Shweta Rai, Judith Angelitta Noronha

**Affiliations:** 1 Department of Fundamentals of Nursing, Manipal College of Nursing, Manipal Academy of Higher Education, Manipal, Karnataka 576104 India; 2 Department of Clinical Psychology, Manipal College of Health Professionals, Manipal Academy of Higher Education, Manipal, Karnataka 576104 India; 3 Department of Obstetrics and Gynaecological Nursing, Manipal College of Nursing, Manipal Academy of Higher Education, Manipal, Karnataka 576104 India

**Keywords:** Adolescent health, Emotional regulations, Well-being, Health promotion, Mental health, Self-control, Psychological adaptation, South asia

## Abstract

**Background:**

Adolescence is a crucial developmental phase marked by significant social, emotional, and psychological transformations. During this period, adolescents frequently face challenges in managing their emotions, which can lead to issues such as irritability, low self-esteem, and mood disorders. As a result, understanding the factors influencing emotional regulation and identifying effective strategies to improve these skills is critical for their overall well-being. This study aims to explore the factors affecting emotional regulation in adolescents and examine the strategies implemented in educational settings to enhance emotional regulation skills among adolescents in South Asian countries.

**Method:**

The “Preferred Reporting Items for Systematic Review and Meta-Analysis (PRISMA)” 2020 was adopted to conduct this systematic review. The literature search was performed via seven databases: PubMed (MEDLINE), Embase (Ovid), Web of Science, CINAHL (EBSCOhost), ProQuest, Scopus and Google scholar. The PRISMA checklist was adopted to report the study findings for transparency and completeness of the study. PROSPERO registration ID is CRD42023467532.

**Results:**

A total of 449 records were initially identified. After screening and full-text review, 24 studies met the inclusion criteria. The included studies were from India (*n* = 14), Nepal (*n* = 2), Pakistan (*n* = 6), Sri Lanka (*n* = 1) and Bangladesh (*n* = 1). The main facilitating factors for Emotional Regulation (ER) were identified as strong peer relationships, parental emotional support, academic motivation, and life skills education. Key hindering factors included authoritarian parenting, parental emotional unavailability, peer victimization, and socioeconomic hardship. Three types of school-based interventions were reported: life skills training, cognitive-emotional regulation programs, and mindfulness-based approaches. Most interventions showed positive short-term outcomes on ER-related behaviors.

**Conclusion:**

ER among South Asian adolescents is shaped by a complex interplay of individual, familial, and contextual factors. While school-based interventions show potential, their limited scope and weak integration into education and health systems constrain their impact. Future efforts must focus on culturally tailored, scalable, and sustainable interventions that are embedded within national adolescent health and education frameworks. Investment in longitudinal research and intersectoral policy responses is essential to address adolescent ER as a core public health priority in the region.

**Supplementary Information:**

The online version contains supplementary material available at 10.1186/s12889-025-24793-8.

## Introduction

Adolescence (10–19 years) is a developmental period in which one’s life is marked by profound physical, emotional and social transformations [1]. During this time, adolescents transition from childhood dependence to adult independence, while navigating challenges related to identity, autonomy, and interpersonal relationships [2]. During this period, the key factor is the ability to regulate emotions as it dictates the mental health, academic performance, and social functioning of the adolescents [3]. Emotional regulation (ER) is broadly defined as the processes through which individuals influence the intensity, duration, and expression of their emotions to achieve adaptive outcomes [4]. Effective ER contributes to resilience, stress management, and positive social adjustment, whereas emotional dysregulation has been associated with a wide range of difficulties, including anxiety, depression, suicidality, self-esteem problems, as well as externalizing behaviors such as anger and aggression [5, 6]. Although ER difficulties manifest across both domains, the existing South Asian evidence base has predominantly examined internalizing outcomes [7]. Accordingly, this review reflects that emphasis while recognizing the broader relevance of ER to externalizing problems as well.

Globally, approximately one in seven adolescents (14%) experiences a mental health conditions, with emotional disorders being most prevalent and often untreated [8]. In South Asia, where adolescents form a considerable proportion of the population, mental health concerns are compounded by region-specific challenges. The region is Home to over 750 million young people aged 15 to 24, and nearly half of all mental health conditions begin by age 14, making early intervention critical [9, 10].

The South Asian Association for Regional Cooperation (SAARC) member states—Afghanistan, Bangladesh, Bhutan, India, Maldives, Nepal, Pakistan, and Sri Lanka, share overlapping public health concerns that disproportionately affect young people [11]. However, country-specific stressors exacerbate emotional vulnerability in adolescents. In India, for example, faces a high prevalence of depression, anxiety, and suicide among adolescents are exacerbated by academic pressure, stigma, and limited access to care [11–13]. Suicide is a leading cause of death among adolescents with over 13,000 cases were reported by the National Crime Records Bureau in 2020 [14]. In Afghanistan and parts of Pakistan, ongoing armed conflict and terrorism have deeply affected adolescent mental health, with widespread poverty, displacement, and erosion of social support systems contributing to heightened emotional distress [10, 11, 15]. Sri Lanka’s history of prolonged civil conflict and ethnic tensions continues to generate intergenerational trauma and weaken institutional trust [16, 17].In the Maldives, adolescents face emotional regulation challenges from violence, family issues, limited education, and social disengagement, compounded by unhealthy lifestyles, substance misuse, and poor mental health support, leading to greater vulnerability to stress, risky behaviours, and long-term psychological harm [18]. Across the region economic crises, poverty, and internal or cross-border migration further exacerbate stress and disrupt education [19]. Migration itself is a recognized determinant of health, often linked to disrupted social support networks, interrupted schooling, and heightened exposure to trauma [19, 20]. Personality traits like resilience [21], and self-esteem [22], along with cognitive factors [3] and hereditary influences [23], affect adolescents’ emotional regulation. Additionally, parenting styles, family dynamics, peer relationships, and socioeconomic status also plays a significant roles in shaping emotional responses and expressions among adolescents [24, 25]. Global and cultural values, for example, socioeconomic status, can also influence adolescent interpretations of emotions and emotional expressions [26].

School-based interventions such as emotion coaching, mindfulness training, and cognitive behavioural therapy (CBT) have shown promise in enhancing ER [27–29]. Social-emotional learning (SEL) programs, when integrated into curricula, provide supportive environments for developing ER skills, while emerging digital tools and mobile apps offer innovative delivery modes [29, 30]. However, evidence from South Asia outside India remains limited, and there is insufficient understanding of how interventions are adapted to local socio-cultural and political contexts [31]. This lack of region-wide evidence justifies the present review, which aims to address a critical gap in literature by synthesizing findings from all SAARC countries. This systematic review focuses exclusively on studies conducted within educational institutions in all eight SAARC countries, with the objectives to: (1) identify factors influencing emotional regulation among adolescents in these settings; (2) examine strategies implemented to enhance ER.

Based on the synthesis, the review will propose health system-aligned recommendations for South Asia, including integrating ER curricula into national education policies, training teachers and school counselors in trauma-informed approaches, establishing school-based screening and referral systems, designing mobile-based support for migrant or conflict-affected youth, and fostering intersectoral collaboration between education, mental health, and social welfare sectors [32, 33].

## Methods

The “Preferred Reporting Items for Systematic Review and Meta-Analysis (PRISMA)” [34] were adopted. The “International Prospective Register of Systematic Reviews (PROSPERO)” database has this systematic review registered under registration number CRD42023467532.

The review question was formulated after a thorough literature search and brainstorming using PICO framework.

I. What are the factors affecting emotional regulation among adolescents in South Asian Countries?
II. What strategies have been implemented in educational settings to enhance emotional regulation skills among adolescents in South Asian Countries?

This study adopted the PICO (Population, Intervention/Exposure, Comparison, Outcome) format, according to the PRISMA [34], for developing the research question Table 1.

**Table 1:** PICO format for formulating the research question

Sl. No	PICO format	Description
1	P (Population)	Adolescents in South Asian countries
2	I (Intervention/Exposure)	Factors affecting emotional regulation and strategies implemented in educational settings to enhance emotional regulation
3	C (Comparison)	(There is no direct comparison in this question, so this part is omitted.)
4	O (Outcome)	Emotional regulation outcomes (such as improved emotional control, emotional well-being, etc.)

In addition to the primary review question, this review also aimed to understand the strategies have been implemented in educational settings to enhance emotional regulation skills among adolescents in South Asian Countries.

### Data sources

A systematic literature search was performed via seven databases: PubMed (MEDLINE) using MeSH terms and synonyms for targeted keywords related to factors; Embase (Ovid) via Emtree; Web of Science; CINAHL (EBSCOhost) via CINHAL Heading; Scopus, Pro Quest and Google scholar. Articles published before the 3rd of December 2024 were included in the study. The search strategies adopted in the databases are given in Supplementary Table 1.

### Inclusion and exclusion criteria

The inclusion and exclusion criteria used in this review are summarized in Table 2.

**Table 2:** Inclusion and exclusion criteria of the studies

Category	Inclusion Criteria	Exclusion Criteria
Population	Adolescents aged 10–19 years based on WHO classification [8]. Studies involving any gender.	Adolescents with special needs, mental illness, or cognitive impairments.
Phenomena of Studies	Studies focusing on factors affecting adolescents’ emotional regulation, methods to improve emotional regulation (educational programs, group interventions, training interventions).	Studies not focusing on factors affecting or methods for enhancing emotional regulation.
Context	Studies conducted in South Asian countries “Afghanistan, Bangladesh, Bhutan, India, Maldives, Nepal, Pakistan, Sri Lanka” [35].	Studies conducted outside South Asia or not in educational institutions.
Outcomes	Primary outcome: Factors affecting emotional regulation. Secondary outcome: Strategies to enhance emotional regulation.	Studies not reporting primary or secondary outcomes related to emotional regulation.
Types of Studies	Original research using mixed or quantitative approaches. Peer-reviewed, English-language studies published until December 3rd, 2024. Study designs: cross-sectional, case-series, time-series, comparative cohort, case-control, RCTs, Non-RCTs. Qualitative research: phenomenology, grounded theory, ethnography, focus groups, qualitative description. Mixed methods accepted if quantitative or qualitative components can be extracted.	Conference proceedings, reports, review papers, editorial letters, article comments, pilot studies, protocol papers.

### Population

Research involving adolescents of any gender aged between 10 and 19 years was taken into consideration. Due to the potential impact on their mental health, reviewers did not include studies involving adolescents with special needs, mental illness, or cognitive impairments.

### Phenomena of studies

The qualitative component of this review focused on studies investigating the factors that impact adolescents’ capacity to control their emotions. The quantitative component of this review examined several methods for enhancing adolescents’ emotional control. These strategies included educational programs, group interventions, and training interventions, all aimed at assisting adolescents in developing better emotional regulation skills.

### Context

This review considers studies conducted in the educational institution of South Asian countries. The following nations are listed by the “South Asian Association for Regional Cooperation: “Afghanistan, Bangladesh, Bhutan, India, Maldives, Nepal, Pakistan, and Sri Lanka” [35].

### Outcomes

The main outcome was to identify factors affecting emotional regulation, whereas the secondary outcome aimed to discover strategies for enhancing emotional regulation.

### Types of studies

This review focused on original research studies (Such as such as cross-sectional, cohort, case-control, RCTs, and Non RCT etc.) that utilized mixed and quantitative approaches. The studies were published in English-language peer-reviewed journals and conducted in South Asia from their inception until 3rd December 2024.

However, the review excluded studies that did not report a primary or secondary outcome. Additionally, conference proceedings, reports, review papers, editorial letters, article comments, protocol paper and pilot studies were not included in this review and were excluded.

### Data extraction

Following the search, all relevant studies was compiled and uploaded to Rayyan software [36], where duplicates were systematically removed. Two independent reviewers (PS and LSG) screened titles, abstracts, and full texts against the inclusion criteria. Any discrepancies were resolved by discussion, with a third reviewer (SR). Data were extracted using an Excel spreadsheet Microsoft 365 Apps for Enterprise version 2504, including study details (author, year, country, design, sample, ER factors, interventions, and outcomes). Reasons for exclusion at the full-text stage were documented.

### Data synthesis strategy

The description and synthesis of the data were conducted based on the main topics, context, and types of sources used. A review was created by narratively synthesizing the retrieved data and presenting it in tables. Summaries of the intervention effects for every study were included whenever possible. A meta-analysis was deemed unsuitable due to lack of homogeneity as the variability of the outcome measures employed in the included studies.

### Quality assessment

Two independent reviewers (PS and LSG) critically assessed the methodological quality of relevant studies using the standard critical assessment checklist created by the Joanna Briggs Institute (JBI) [37]. The JBI critical appraisal checklist was used to evaluate observational and quasi-experimental studies as well as analytical cross-sectional studies [38] The methodological quality of the 24 included studies—comprising 21 cross-sectional studies appraised using the *JBI appraisal checklist for analytical cross-sectional studies* [38], two quasi experimental studies assessed with the *JBI Checklist for Quasi-Experimental Studies* and one mixed methods study evaluated using the Mixed Methods Appraisal Tools Version 2018 [39] the data is depicted in the (Supplementary Table 2). For the JBI tools, each item within the checklist was rated either as “Yes”, “No”, “Unclear”, or “Not Applicable”. The derived quality score here was a proportion of “Yes” responses. Accordingly, scores of ≥ 75% were considered high quality, those of 50–74% were considered moderate quality, and those of < 50% were considered low quality.

## Results

**Fig. 1:**
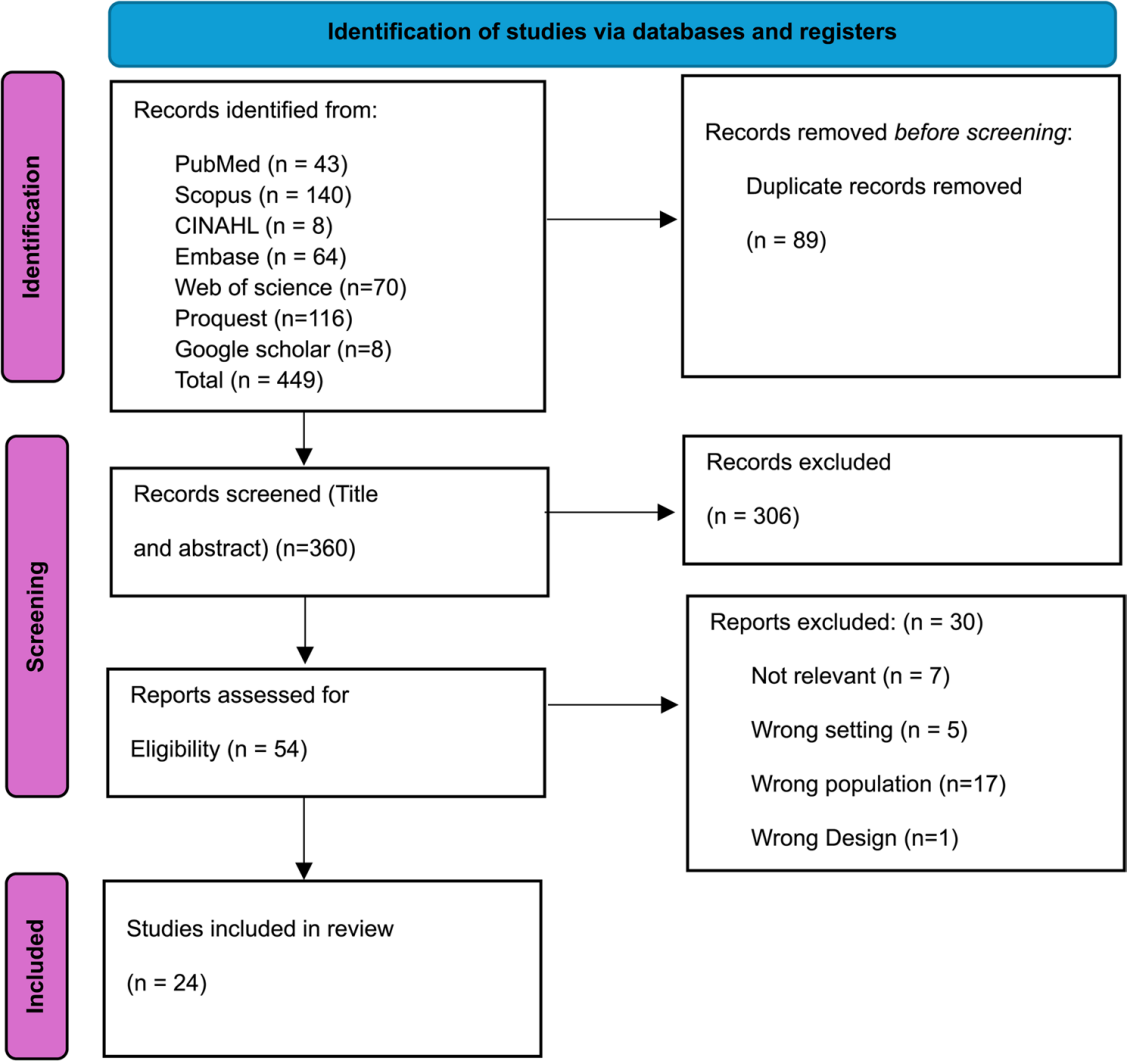
PRISMA flowchart of the article selection process

The process of study selection can be seen in the PRISMA flowchart. A total of 449 articles were identified through database searching across PubMed, Scopus, CINAHL, Embase, Web of Science, ProQuest, and Google Scholar, and uploaded to Rayyan Software, and 89 of these records were found to be duplicates and eliminated from the review. After removing duplicates, 360 articles were eligible for title and abstract screening, and 306 records were excluded after the titles and abstracts were screened. Therefore, the remaining 54 articles were eligible for full-text screening. Thus, after the full-text screening, 24 articles were included in this systematic review (Fig. 1).

### Study characteristics

The 24 articles included in this study, with the majority being cross-sectional (*n* = 21), quasi-experimental (*n* = 2), and mixed method studies (*n* = 1). The study characteristics of each of the articles included in this systematic review are listed in supplementary file Table 3, which includes the study’s geographical setting, sample size, and participant demographics (age, gender). Among the 24 studies, 14 (58.3%) were conducted in India, 6 (25%) in Pakistan, 2 (8.3%) in Nepal, and 1 (4.2%) each in Bangladesh and Sri Lanka. Sample sizes varied widely from 10 [40] to 1,500 [41]. Commonly used emotional regulation measures included the Difficulties in Emotion Regulation Scale (DERS), Emotion Regulation Questionnaire (ERQ).

The age distribution for participants would be described in different studies in means and standard deviation, ranges, and while others provided minimal details such as the range. The youngest age across studies is ten years [42], while the oldest is nineteen years [25, 26, 41, 43–46].

### Factors that influence emotional regulation among adolescents

**Fig. 2:**
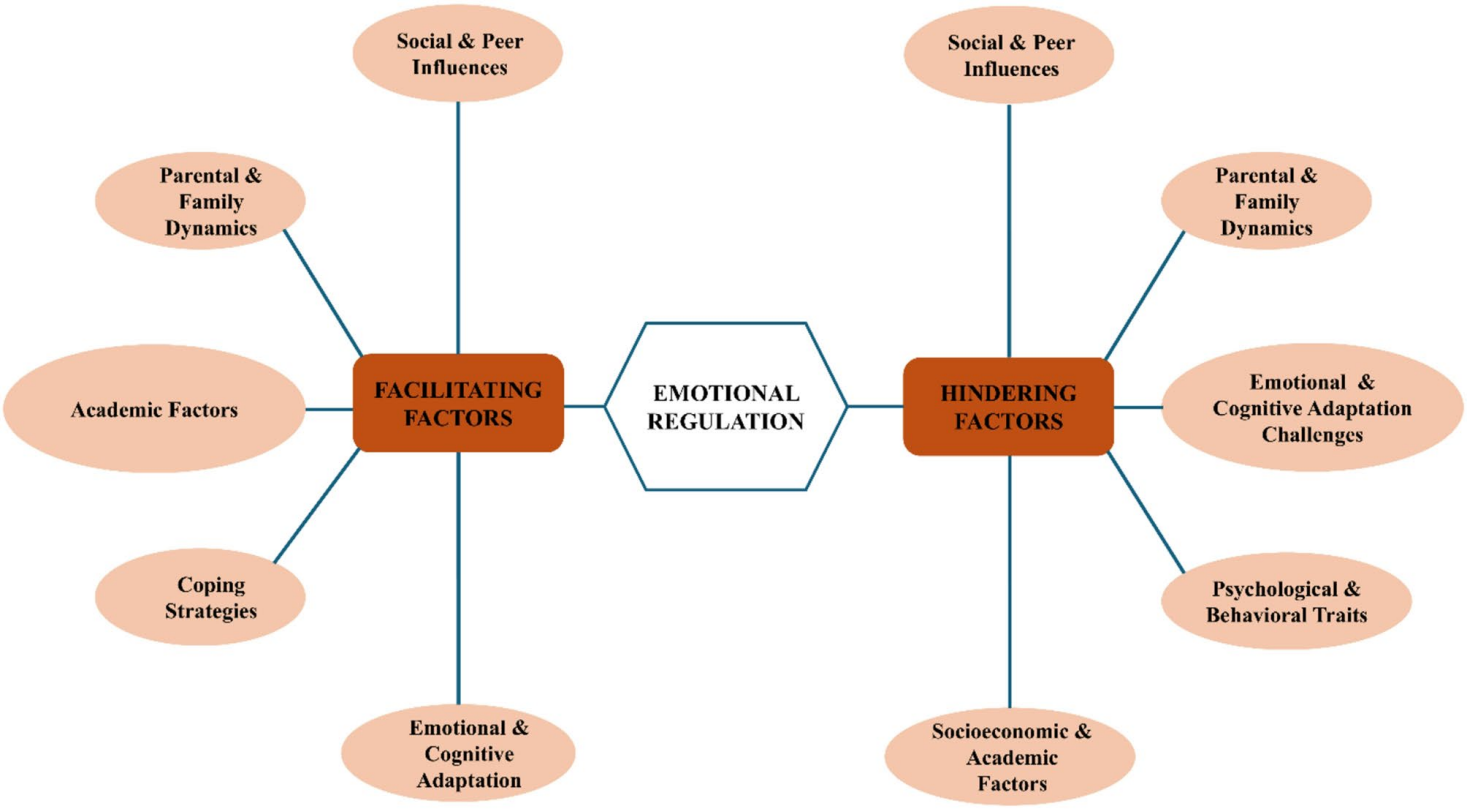
Categorization of facilitating and hindering factors influencing adolescent emotional regulation identified in included studies

There are multiple factors that significantly influence emotional regulation processes in adolescents can be categorized into two distinct groups: *facilitating factors* that enhance emotional regulation capabilities and *hindering factors* that impede these processes. These facilitating and hindering factors have been divided into five different groups to provide more clarity (Fig. 2). Additionally, there was a significant variation in the components, analysis methods, outcomes assessments, and study design. As a result, tables and figures were used to support the descriptive presentation of the data. Table 3 provides a summary of the facilitating and hindering factors.

**Table 3:** Summary of facilitating and hindering factors affecting adolescent emotional regulation across included studies

Facilitating Factors		Hindering Factors	
Social and Peer Influences	• Positive Peer Relationships [43] • Higher level of social support from close friends [46] • Supportive environments [47] • Positively influence peer relationships [47] • Positive romantic relationship qualities [48] • Social Impact [49] • Gratification of Needs[49] • Gender differences [26] • Handling relationships [26]	Social and Peer Influences	• Bullying and Victimization [43] • Rejection Sensitivity [43] • Negative peer interactions [47] • Cyber victimization [50] • Cyber aggression[50] • Negative romantic relationship qualities [48] • Increased health-risk behaviors [51,52]
Parental and Family Dynamics	• Positive maternal expressed emotions [45] • Strong parent-child attachment quality [53] • Parental emotion socialization [42] • Intergenerational Support [25] • Perceived positive parenting practices [52] • Joint family systems [54] • Supportive Parental Responses [42] • Supportive maternal behaviors [45]	Parental and Family Dynamics	Parenting Styles: • Authoritarian Parenting [24, 25] • Negative Parenting styles [52] • Punitive Parental Responses [42] • Non-supportive parenting behaviors [24] • Over-expectations [49] • Influence of Family Environment [49] • Nuclear family systems [54] Maternal and Parental Emotional Factors: • Mother Overprotection [44] • Critical comments and hostility from mothers [45] • Negative maternal emotional expressions [45] • Parental ER difficulties [53] • Perception of non-acceptance of emotional responses by parents [49]
Emotional and Cognitive Adaptation	• Cognitive Reappraisal [25, 48, 50, 55] • Expressive Suppression [50] • Emotional Self-Regulation [49] • Empathy [26] • Resilience [55] • Prosocial Behavior [55]	Emotional and Cognitive Adaptation Challenges	• Emotion-focused coping [56] • Expression suppression [25, 48, 55] • Non-acceptance of emotional responses [41, 46] • Emotional dysregulation [44] • Lack of emotional awareness [46, 57] • Lack of emotional clarity [41, 46] • Negative self-image [44] • Impulse control difficulties [41, 57] • Limited access to emotion regulation strategies [41, 57] • Feelings of Frustration [49]
Coping Strategies	• Effective Self-Regulatory Strategies [43] • Problem-focused coping strategies [56] • Coping strategies to manage anger effectively[49] • Spirituality [56] • Yoga Practice [58]	Psychological and Behavioral Traits	• Negative thinking [43] • Psychoticism [26] • Antisocial behavior [55] • Criminal propensity [26] • Exposure to traumatic events [56]
Academic Factors	• Academic motivation and performance [57] • Adolescent School Engagement [24]	Socioeconomic and Academic Factors	• Lower Socio-Economic Status (SES) [53] • Poor academic performance [57] • Difficulties in engaging in goal-directed activity [57]

### Strategy to enhance emotional regulation

There are multiple strategies have been identified to enhance emotional regulation which include life skills training programs introduced by WHO [59], cognitive emotional regulation intervention strategies [60], and the school-based emotion regulation prevention intervention (READY-Nepal) [40] (Table 4).

**Table 4:** Description of the interventional studies included in the systematic review

Citation	Country	Intervention name	Duration	Delivered by	Delivery format	Theoretical basis	Outcome assessment	Follow up status	Key Outcomes
G.B.Chaudhari, 2021 [59]	India	Life skills training programs	60 h (2 h X 30 session)	Not Mentioned	Classroom discussions, Group tasks, educational games, role plays, brainstorming and storytelling.	WHO Life Skills framework	Emotional Competence Scale	Post testing after intervention no follow up	Significant improvement in emotional competence (pre = 88.07, post = 98.89, t = 5.04, *p* < 0.01). Girls scored higher than boys both at pre-test and post-test.
Agarwal et al., 2023 [60]	India	Cognitive Emotional Regulation Intervention	20 sessions X 80 min online	Buddy counsellor	Online sessions with parent involvement	Combining attachment theory, Emotional regulation in Adolescence, group Dialectical Behavioral Therapy skills, and group schema therapy techniques.	CERQ	Post test only	Significant increases in adaptive strategies (acceptance, positive refocusing, positive reappraisal, putting things into perspective and refocus on planning); significant decreases in maladaptive strategies (rumination, catastrophizing, self-blame and other blame.); at *p* < 0.01 level of significance.
Ramaiya et al., 2022 [40]	Nepal	School-Based Emotion Regulation Prevention Intervention (READY-Nepal)	8 Group session X (50 min each over 4 weeks)	By 2 local research assistants, US doctoral student under the supervision of experienced DBT clinicians	School-based, classroom group sessions (gender segregated)	Dialectical Behavior Therapy (DBT) with cultural adaptation to Nepali context	DERS, DBT-WCCL, BAI, CPSS, CFI, Resilience scale, Suicidal ideation	immediate post intervention follow up	No significant group effects on emotion regulation, coping, PTSD, or resilience, though slight non-significant improvements were observed. Anxiety showed a significant gender × time effect, with females improving more than males. Functional impairment significantly decreased across both groups (*p* = 0.004). Suicidal ideation reduced by 23% in the intervention group (notably among females), while remaining unchanged or increasing in controls.

*DERS* Difficulties in Emotion Regulation Scale, *DBT-WCCL* Dialectical Behavior Therapy Ways of Coping Checklist, *BAI* Beck Anxiety Inventory, *CPSS* Child version of the Posttraumatic Symptom Scale, *CFI* Child functioning impairment scale, *CERQ* Cognitive Emotion Regulation Questionnaire

## Discussion

This systematic review synthesizes evidence on the multifactorial determinants and intervention strategies for ER among adolescents in South Asia, a region marked by socio-economic disparity, academic pressure, and significant mental health challenges. In line with the study objectives, this discussion is organized in two parts first, the factors influencing emotional regulation among adolescents and second, the strategies implemented to enhance ER. This followed by gaps, and policy implications.

### Factors influencing emotional regulation

#### Cultural and familial influences

Consistent with global evidence, this review confirms that the role of supportive, warm and autonomy encouraging parenting, is linked with better adolescent ER, and adjustment, whereas harsher or controlling practices are associated with ER difficulties [24, 25, 42, 44, 52, 53]. At the same time, broader regional literature indicates that cultural norms such as emotional suppression, authoritarian parenting, and stigma around mental health constrain healthy emotional development [61–64]. Unlike Western contexts where emotional expression and autonomy are emphasized, South Asian societies often encourage restraint, particularly within patriarchal family structures [65–67]. Gender expectations further complicate this picture: boys are often discouraged from expressing vulnerability, while girls are socialized into obedience and modesty, limiting emotional agency [68].

#### Peer and social influences

Adolescents’ ER is shaped by their social worlds: systematic review evidence shows adverse peer experiences (e.g., victimization, cyberbullying, rejection) are negatively associated with ER at behavioural and neural levels, underscoring the value of positive peer climates [43, 46, 47, 49]. Consistent with this, studies show that positive peer interactions foster emotional and social growth, whereas exclusion and peer pressure contribute to difficulties [69]. Peer dynamics, including bystander behavior, further shape bullying trajectories and regulation capacities [70]. Collectively, this highlights the decisive role of peer environments in shaping adolescents’ emotional regulation across contexts.

#### Cognitive and emotional traits

Adolescents’ cognitive and emotional traits play a central role in shaping their emotion regulation (ER) capacity within educational settings. Evidence from high-income countries shows a reciprocal relationship between ER and classroom engagement: students with low emotional awareness and clarity demonstrate reduced behavioural and emotional engagement, while greater engagement enhances access to adaptive ER strategies and supports improved peer and teacher relationships [71].

### Strategies implemented to enhance emotional regulation

The interventions identified in this review include life skills training programs, cognitive-emotional regulation modules, and mindfulness/DBT-based approaches such as the READY-Nepal model [40]. Across contexts, these interventions demonstrated short-term improvements in emotional awareness, coping strategies, and interpersonal skills. For example, G.B.Chaudhari, 2021 [59] reported significant gains in emotional competence following a structured life skills program. Agarwal et al., 2023 [60] demonstrated reductions in maladaptive emotional responses through online DBT- and schema therapy–based modules. However, the overall evidence base remains limited in scope, with most interventions being small-scale pilots, lacking rigorous designs, standardized assessment tools, and long-term follow-up. Beyond the three South Asian studies included in this review, global evidence consistently highlights the effectiveness of school-based life skills and social-emotional learning (SEL) programs in enhancing adolescents’ emotional regulation and psychosocial outcomes. A meta-analysis of 213 school-based programs across high- and middle-income countries demonstrated significant improvements in social-emotional skills, behavior, and academic performance [28]. Similarly, a review of mindfulness-based interventions showed reductions in stress and improvements in emotional regulation among adolescents [27, 72]. Evidence for Dialectical Behavior Therapy (DBT) adapted programs in adolescent populations also demonstrates reductions in self-harm and improved coping [73]. While findings from South Asia remain limited to small-scale feasibility studies, evidence from high-income countries shows stronger outcomes often underpinned by robust infrastructure, systematic teacher training, and policy-level integration of SEL and mental health promotion. Large-scale SEL programs in high income countries benefit from sustained financing and longitudinal evaluation features largely absent in low-resource South Asian contexts. This contrast underscores the implementation and scalability challenges that low- and middle-income countries face, despite equally pressing needs for ER interventions [29, 74]. Taken together, these findings suggest that while South Asian interventions remain small-scale and under-evaluated, they are broadly consistent with international trends showing the promise of life skills, cognitive-emotional, and mindfulness-based approaches for strengthening adolescent ER.

#### Gaps in implementation and research

Despite the relevance of school-based ER interventions, implementation remains uneven across South Asia. Schools in South Asia rarely embed ER initiatives into curricula, and teachers often lack training in psychosocial support [75, 76]. Interventions requiring specialized psychological expertise, such as DBT, face feasibility challenges in under-resourced settings where trained professionals are scarce [60]. Broader literature highlights that while digital platforms (e.g., mobile apps, tele-counselling) could expand access, the digital divide in rural and low-income communities creates inequities in uptake [77]. Furthermore, most available evidence does not adequately disaggregate by gender, caste, or rural–urban location, leaving gaps in understanding how structural inequities shape ER outcomes. Another important research gap relates to the distinction between internalizing (e.g., anxiety, depression) and externalizing (e.g., aggression, conduct problems) emotional regulation difficulties, which is well established in global literature [78–80]. However, most South Asian studies included in this review did not explicitly apply this framework, limiting our ability to differentiate how ER processes manifest across these domains. Future studies should incorporate validated measures of both internalizing and externalizing outcomes to allow for more nuanced analysis and to better inform context-specific interventions.

#### Policy recommendations

From a policy perspective, integrating ER into existing adolescent health and education frameworks is both feasible and necessary. India’s Rashtriya Kishor Swasthya Karyakram (RKSK), for instance, offers a potential entry point for embedding ER-focused interventions within adolescent-friendly health services [81, 82]. In Sri Lanka, a national Guidance and Counselling framework exists, but implementation is uneven beyond urban schools [83, 84]. In Nepal, the National Mental Health Strategy and Action Plan (2020) emphasizes integrating mental health promotion into the school curriculum and training teachers and school health workers in child and adolescent mental health [85]. The government has also introduced the pilot initiatives such as “One School, One Nurse” are promising steps, actual systematic integration of mental health activities and sustainable, nationwide implementation in schools is still lacking. The principal gaps are insufficient scaled training, limited deployment of school health workers, and uneven coverage between urban and rural areas [86, 87].

However, without dedicated financing, systematic training, and monitoring mechanisms, such opportunities remain underutilized. Expert commentary emphasizes the need for cross-sectoral collaboration between ministries of health, education, and child development to mainstream ER in adolescent well-being strategies [88]. Hybrid delivery models—such as culturally adapted digital tools [89] combined with offline community-based initiatives like peer-support groups and parental emotional literacy programs [90] could address both access and contextual relevance. Public campaigns to normalize emotional expression and reduce stigma are also critical [31]. Finally, interventions must extend beyond individual coping skills to address structural determinants such as poverty, gender inequality, and educational inequity [91, 92]. Experiences from high-income countries demonstrate that embedding ER into education and health systems is most effective when supported by strong institutional infrastructure, teacher training, and long-term investment. For South Asia and other low-resource settings, adapting these lessons into scalable, culturally grounded, and equity-focused models will be essential [29]. Overall, embedding ER into adolescent health and education policies in South Asia requires sustained political will, cross-sectoral collaboration, and investment in scalable, culturally relevant models. Unless these recommendations are translated into actionable policy, adolescent emotional well-being will remain neglected despite being central to human capital development in the region.

### Limitations

This review offers important insights into emotional regulation among adolescents in South Asia, but several limitations must be acknowledged. First, the review was restricted to studies published in English and peer-reviewed sources, which may have excluded relevant research available in local languages or unpublished gray literature, thereby introducing potential publication bias. Second, the included studies were highly heterogeneous in terms of design, interventions, and outcome measures, which precluded a meta-analysis and limited the ability to generate pooled effect estimates. Third, although efforts were made to minimize bias during study selection and data extraction through double screening, the possibility of reviewer bias cannot be entirely excluded, as inter-rater reliability was not formally calculated. Fourth, although the search strategy was designed to capture a wide range of constructs related to emotional regulation, it did not explicitly include the categories of “internalizing” and “externalizing” problems. As a result, some studies framed primarily within these domains may not have been retrieved. Fifth, many of the included interventions were pilot or small-scale studies with limited follow-up, restricting the assessment of long-term effectiveness and sustainability. Sixth, contextual and structural determinants such as gender inequities, rural-urban differences, and cultural stigma around mental health were not consistently analysed across the included studies, in addition, most of the included studies were cross-sectional, which constrains causal inference and underscores the need for more longitudinal and experimental designs to examine changes in emotional regulation over time.

### Implications

In future research, intervention studies focusing on adolescent’s emotions should consider the factors that hinder emotional regulation, while also emphasizing strategies that are feasible within public health systems. Advances in technology have facilitated the delivery of emotion regulation training in resource-limited settings, and such tools can be integrated into schools, community centres, and health facilities where adolescents feel comfortable expressing themselves. Although modules have been prepared for implementation in schools, the lack of facilities and resources often prevents their regular delivery, underscoring the need for continuous monitoring systems to ensure fidelity and sustainability. Furthermore, persistent societal stigma around mental health continues to limit open engagement, highlighting the importance of awareness campaigns and community involvement. Embedding these approaches into existing public health initiatives such as school health programs, teacher and school nurse training, and community-based adolescent services can enhance scalability and sustainability. Finally, an inter-institutional approach among researchers, educators, policymakers, and other stakeholders is essential to ensure evidence-based, contextually relevant, and sustainable outcomes for adolescent well-being.

## Conclusion

Emotional regulation (ER) among South Asian adolescents is shaped by multiple influences, including parenting practices, peer relationships, cognitive traits, academic engagement, socioeconomic background, and cultural norms. This review also highlighted strategies such as life skills programs, cognitive–emotional strategies, and mindfulness/DBT-based approaches demonstrate short-term benefits, though evidence remains limited and fragmented. Integrating ER into existing adolescent health and education frameworks is both feasible and necessary, for instance through short, structured modules embedded in weekly school schedules and primary healthcare visits, with teachers and school counsellors serving as frontline delivery agents and psychologists providing referral support for high-risk cases. Hybrid models that combine mobile applications featuring mood tracking, interactive skill-building, and peer forums with offline supports such as classroom mindfulness exercises or community-based youth groups have shown promise in other low-resource contexts and could be adapted to South Asia. Building local capacity through teacher training and peer facilitators offers another pathway to scale interventions in settings where mental health professionals are scarce.

At the same time, advancing the evidence base will require longitudinal and experimental research designs, standardized tools capturing both internalizing and externalizing dimensions of ER, and cost-effectiveness evaluations to inform sustainable implementation. By embedding culturally sensitive ER initiatives into school and health systems, supported by scalable delivery mechanisms and rigorous research, South Asian countries can address a critical gap in adolescent mental health and strengthen their broader human capital development.

## Supplementary Information

Supplementary Material 1.

Supplementary Material 2.

## Data Availability

Data was provided within the manuscript or supplementary information files.

## Abbreviations

ER: Emotional regulation
PRISMA: Preferred reporting items for systematic review and meta-analysis
LMIC: Low middle-income countries
WHO: World health organisation
SAARC: South asian association for regional cooperation
RCT: Randomised controlled trail
DBT: Dialectical behavior therapy
