# Supplementary material for: Factors affecting and the strategies to enhance emotional regulation among adolescents in South Asian countries: a systematic review
Source: BMC Public Health. 2025 Oct 14;25:3485. doi: 10.1186/s12889-025-24793-8 (PMC12523010; doi:10.1186/s12889-025-24793-8)
Supplement: Supplementary file 2 — Supplementary Material 2. [file 12889_2025_24793_MOESM2_ESM.docx]

**Supplementary Table 1**

*Search strategy for PubMed (MEDLINE), Embase (Ovid), Web of Science, CINAHL (EBSCOhost), and Scopus.*

| **Databases** | **Search Terms** | **No. Of Study** |
| --- | --- | --- |
| PUBMED | (((((((((((((((((((((Adolescent[MeSH Terms]) OR (Child[MeSH Terms])) OR (Students[MeSH Terms])) OR (adolescen*[Title/Abstract])) OR ("Adolescents female"[Title/Abstract])) OR ("Adolescents Male"[Title/Abstract])) OR (Teen*[Title/Abstract])) OR (Youth*[Title/Abstract])) OR (Minor*[Title/Abstract])) OR (Child*[Title/Abstract])) OR ("School Children"[Title/Abstract])) OR ("School Enrollment"[Title/Abstract])) OR ("School going Child*"[Title/Abstract])) OR (Young[Title/Abstract])) OR (Juvenile[Title/Abstract])) OR (Student*[Title/Abstract])) OR ("Young people"[Title/Abstract])) OR ("young person"[Title/Abstract]) AND (english[Filter])) AND ((((((((((Emotional regulation[MeSH Terms]) OR ("Emotion Regulation"[Title/Abstract])) OR ("Emotional regulat*"[Title/Abstract])) OR ("Emotional modulat*"[Title/Abstract])) OR ("Emotion Self- Regulation"[Title/Abstract])) OR ("Emotional Self- Regulation"[Title/Abstract])) OR ("emotional skills"[Title/Abstract])) OR ("emotional competences"[Title/Abstract])) OR ("emotion dysregulation"[Title/Abstract])) OR ("emotional Dysregulat*"[Title/Abstract]) AND (english[Filter]))) AND ((((((((((strateg*[Title/Abstract]) OR (Tactics[Title/Abstract])) OR ("Plan of action"[Title/Abstract])) OR (Program*[Title/Abstract])) OR (Methods[MeSH Terms])) OR (Method*[Title/Abstract])) OR (Intervention[Title/Abstract])) OR (Scheme*[Title/Abstract])) OR (Approach*[Title/Abstract])) OR (((((Elements[MeSH Terms]) OR (Element*[Title/Abstract])) OR (Characteristics[Title/Abstract])) OR (Component*[Title/Abstract])) OR (Factor*[Title/Abstract])) AND (english[Filter]))) AND ((((((((((((((Schools[MeSH Terms]) OR (School*[Title/Abstract])) OR ("Educational institu*"[Title/Abstract])) OR ("Primary School*"[Title/Abstract])) OR ("Secondary School*"[Title/Abstract])) OR (institu*[Title/Abstract])) OR (Univ*[Title/Abstract])) OR ("Pre- Univ*"[Title/Abstract])) OR (College*[Title/Abstract])) OR ("High Secondary"[Title/Abstract])) OR (Elementary*[Title/Abstract])) OR ("high school*"[Title/Abstract])) OR ("Middle school*"[Title/Abstract])) OR (Education*[Title/Abstract]) AND (english[Filter]))) AND (Afghanistan OR bangladesh OR Bhutan OR India OR Maldives OR Nepal OR Pakistan OR "Sri lanka" OR "South asian countries" AND (english[Filter])) | 43 |
| Web of Science | TI=("Emotion Regulation" OR "Emotional regulat*" OR "Emotional modulat*" OR "Emotion Self-Regulation" OR "Emotional Self-Regulation" OR "emotional skills" OR "emotional competences" OR "Emotion Dysregulation" OR "Emotional Dysregulat*") OR AB=("Emotion Regulation" OR "Emotional regulat*" OR "Emotional modulat*" OR "Emotion Self-Regulation" OR "Emotional Self-Regulation" OR "emotional skills" OR "emotional competences" OR "Emotion Dysregulation" OR "Emotional Dysregulat*") AND TI=(strateg* OR Tactics OR "Plan of action" OR Program* OR Method* OR Intervention OR Scheme* OR Approach*) OR AB=(strateg* OR Tactics OR "Plan of action" OR Program* OR Method* OR Intervention OR Scheme* OR Approach*) OR TI=(Element* OR Characteristics OR Component* OR Factor*) OR AB=(Element* OR Characteristics OR Component* OR Factor*) AND TI=(Adolescent OR Child OR Students OR adolescen* OR Adolescents OR "Female Adolescents" OR "Male Teen*" OR Youth* OR Minor* OR Child* OR "School Children" OR "School Enrollment" OR "School Going Child*" OR "Young Juvenile Student*" OR "young people" OR "young person") OR AB=( Adolescent OR Child OR Students OR adolescen* OR Adolescents OR "Female Adolescents" OR "Male Teen*" OR Youth* OR Minor* OR Child* OR "School Children" OR "School Enrollment" OR "School Going Child*" OR "Young Juvenile Student*" OR "young people" OR "young person") AND TI=(School* OR "Educational institu*" OR "Primary School*" OR "Secondary School*" OR "Institu*" OR "Univ*" OR "Pre-Univ*" OR "College*" OR "Higher secondary" OR "elementary*" OR "high school*" OR "middle school*" OR education*) OR AB=(School* OR "Educational institu*" OR "Primary School*" OR "Secondary School*" OR "Institu*" OR "Univ*" OR "Pre-Univ*" OR "College*" OR "Higher secondary" OR "elementary*" OR "high school*" OR "middle school*" OR education*) AND ALL=(Afghanistan OR Bangladesh OR Bhutan OR India OR Maldives OR Nepal OR Pakistan OR "Sri Lanka" OR "South Asian Countries") | 70 |
| Scopus | ( ( TITLE-ABS-KEY ( "Emotion Regulation" OR "Emotional regulat*" OR "Emotional modulat*" OR "Emotion Self-Regulation" OR "Emotional Self-Regulation" OR "emotional skills" OR "emotional competences" OR "Emotion Dysregulation" OR "Emotional Dysregulat*" ) ) AND ( TITLE-ABS-KEY ( school* OR "Educational institu*" OR "Primary School*" OR "Secondary School*" OR "Institu*" OR "Univ*" OR "Pre-Univ*" OR "College*" OR "Higher secondary" OR "elementary*" OR "high school*" OR "middle school*" OR education* ) ) AND ( TITLE-ABS-KEY ( adolescent OR child OR students OR adolescen* OR adolescents OR "Female Adolescents" OR "Male Teen*" OR youth* OR minor* OR child* OR "School Children" OR "School Enrollment" OR "School Going Child*" OR "Young Juvenile Student*" OR "young people" OR "young person" ) ) AND ( ( TITLE-ABS-KEY ( strateg* OR tactics OR "Plan of action" OR program* OR method* OR intervention OR scheme* OR approach* ) ) OR ( TITLE-ABS KEY ( element* OR characteristics OR component* OR factor* ) ) ) ) AND ( ALL ( afghanistan OR bangladesh OR bhutan OR india OR maldives OR nepal OR pakistan OR "Sri Lanka" OR "South Asian Countries" ) ) AND ( LIMIT-TO ( LANGUAGE , "English" ) ) AND ( LIMIT-TO ( AFFILCOUNTRY , "Bangladesh" ) OR LIMIT-TO ( AFFILCOUNTRY , "India" ) OR LIMIT-TO ( AFFILCOUNTRY , "Nepal" ) OR LIMIT-TO ( AFFILCOUNTRY , "Pakistan" ) OR LIMIT-TO ( AFFILCOUNTRY , "Sri Lanka" ) ) | 140 |
| CINHAL | ( ( TITLE-ABS-KEY ( "Emotion Regulation" OR "Emotional regulat*" OR "Emotional modulat*" OR "Emotion Self-Regulation" OR "Emotional Self-Regulation" OR "emotional skills" OR "emotional competences" OR "Emotion Dysregulation" OR "Emotional Dysregulat*" ) ) AND ( TITLE-ABS-KEY ( school* OR "Educational institu*" OR "Primary School*" OR "Secondary School*" OR "Institu*" OR "Univ*" OR "Pre-Univ*" OR "College*" OR "Higher secondary" OR "elementary*" OR "high school*" OR "middle school*" OR education* ) ) AND ( TITLE-ABS-KEY ( adolescent OR child OR students OR adolescen* OR adolescents OR "Female Adolescents" OR "Male Teen*" OR youth* OR minor* OR child* OR "School Children" OR "School Enrollment" OR "School Going Child*" OR "Young Juvenile Student*" OR "young people" OR "young person" ) ) AND ( ( TITLE-ABS-KEY ( strateg* OR tactics OR "Plan of action" OR program* OR method* OR intervention OR scheme* OR approach* ) ) OR ( TITLE-ABS-KEY ( element* OR characteristics OR component* OR factor* ) ) ) ) AND ( ALL ( afghanistan OR bangladesh OR bhutan OR india OR maldives OR nepal OR pakistan OR "Sri Lanka" OR "South Asian Countries" ) ) AND ( LIMIT-TO ( AFFILCOUNTRY , "Bangladesh" ) OR LIMIT-TO ( AFFILCOUNTRY , "India" ) OR LIMIT-TO ( AFFILCOUNTRY , "Nepal" ) OR LIMIT-TO ( AFFILCOUNTRY , "Pakistan" ) OR LIMIT-TO ( AFFILCOUNTRY , "Sri Lanka" ) ) AND ( LIMIT-TO ( LANGUAGE , "English" ) ) | 8 |
| Embase | ( (MM "Emotional Regulation") OR "emotional regulation" OR (MM "Self Regulation") OR (MM "Emotional Maturity") ) AND ( "strategies" OR (MM "Learning Methods") OR (MM "Sex Factors") OR (MM "Geographic Factors") OR (MM "Biological Factors") OR (MM "Race Factors") OR (MM "Economic Factors") OR "factors" OR (MM "Socioeconomic Factors") OR (MM "Age Factors") OR (MM "Social Factors") OR (MM "Sociodemographic Factors") ) AND ( MM "Students") OR "adolescents" OR (MM "Adolescence") OR (MM "Child Development: Adolescence (12-17 Years) (Iowa NOC)") OR (MM "Adolescent Behavior") OR (MM "Adolescent Health") OR (MM "Adolescent Development") ) AND ( (MM "Schools") OR "schools" OR (MM "Schools, Middle") OR (MM "Schools, Secondary") OR (MM "Schools, Elementary") ) AND ( TX afghanistan OR TX bhutan OR TX nepal OR TX india OR TX sri lanka OR TX bangladesh OR TX maldives OR TX pakistan OR TX south asia OR TX south asian countries ) | 64 |

**Supplementary Table 2**

*Methodological quality of included studies*

| **JBI Checklist for Observational Studies** | | | | | | | | | | |
| --- | --- | --- | --- | --- | --- | --- | --- | --- | --- | --- |
| **Study Citation** | **1. Were the criteria for inclusion in the sample clearly defined?** | **2. Were the study subjects and the setting described in detail?** | **3. Was the exposure measured in a valid and reliable way?** | **4. Were objective, standard criteria used for measurement of the condition?** | **5. Were confounding factors identified?** | **6. Were strategies to deal with confounding factors stated?** | **7. Were the outcomes measured in a valid and reliable way?** | **8. Was appropriate statistical analysis used?** | **Overall** | **Weight (%)** |
| (Batool & Shafiq, 2022) | 1 | 1 | 1 | 1 | 1 | 0 | 1 | 1 | + | 87.5% |
| (Uddin & Rahman, 2022) | 0 | 1 | 1 | 1 | 1 | 0 | 1 | 1 | + | 75% |
| (Iqbal et al., 2023) | 1 | 1 | 1 | 1 | 1 | 0 | 1 | 1 | + | 87.5% |
| (Akhtar & Bano, 2023) | 1 | 1 | 1 | 1 | 0 | 0 | 1 | 1 | + | 75% |
| (Gunawarden & Schuck, 2021) | 1 | 1 | 1 | 1 | 0 | 0 | 1 | 1 | + | 75% |
| (P. Singh & Singh, 2013) | 0 | 1 | 1 | 1 | 0 | 0 | 1 | 1 | - | 62.5% |
| (Tanveer et al., 2023) | 0 | 1 | 1 | 1 | 1 | 0 | 1 | 1 | + | 75% |
| (R. Singh et al., 2020) | 1 | 1 | 1 | 1 | 1 | 0 | 1 | 1 | + | 87.5% |
| (Natso et al., 2018) | 1 | 1 | 1 | 1 | 0 | 0 | 1 | 1 | + | 75% |
| (Raval et al., 2018) | 0 | 1 | 1 | 1 | 1 | 0 | 1 | 1 | + | 75% |
| (Janjhua et al., 2020) | 1 | 1 | 1 | 1 | 0 | 0 | 1 | 1 | + | 75% |
| (Kaul et al., 2019) | 1 | 1 | 1 | 1 | 0 | 0 | 1 | 1 | + | 75% |
| (Saleem & Gul, 2018) | 0 | 1 | 1 | 1 | 0 | 0 | 1 | 1 | - | 62.5% |
| (Zafar et al., 2021) | 0 | 1 | 1 | 1 | 1 | 1 | 1 | 1 | + | 87.5% |
| (Borthakur, 2019) | 0 | 1 | 1 | 1 | 0 | 0 | 1 | 1 | - | 62.5% |
| (P. Singh & Singh, 2022) | 1 | 1 | 1 | 1 | 0 | 0 | 1 | 1 | + | 75% |
| (Dash & Verma, 2017) | 0 | 1 | 1 | 1 | 0 | 0 | 1 | 1 | - | 62.5% |
| (G. Singh, 2022) | 1 | 1 | 1 | 1 | 1 | 0 | 1 | 1 | + | 87.5% |
| (Dubey et al., 2024) | 1 | 1 | 1 | 1 | 1 | 1 | 1 | 1 | + | 100% |
| (Sia & Aneesh, 2024) | 0 | 1 | 1 | 1 | 1 | 0 | 1 | 1 | + | 100% |
| (P. Singh, 2024) | 1 | 1 | 1 | 1 | 1 | 1 | 1 | 1 | + | 100% |

Scoring: (+) Low Risk, (-) Moderate Risk (X) High Risk

| **JBI Checklist for Quasi-Experimental Studies** | | | | | | | | | | | |
| --- | --- | --- | --- | --- | --- | --- | --- | --- | --- | --- | --- |
| **Study Citation** | **Is it clear in the study what is the “cause” and what is the “effect” (i.e. there is no confusion about which variable comes first)?** | **Was there a control group?** | **Were participants included in any comparisons similar?** | **Were the participants included in any comparisons receiving similar treatment/care, other than the exposure or intervention of interest?** | **Were there multiple measurements of the outcome, both pre and post the intervention/exposure?** | **Were the outcomes of participants included in any comparisons measured in the same way?** | **Were outcomes measured in a reliable way?** | **Was follow-up complete and if not, were differences between groups in terms of their follow-up adequately described and analyzed** | **Was appropriate statistical analysis used?** | **Overall** | **Weight (%)** |
| (G.B.Chaudhari, 2021) | 1 | 0 | 1 | 1 | 1 | 1 | 1 | 0 | 1 | + | 77.7% |
| (Agarwal et al., 2023) | 1 | 0 | 1 | 1 | 1 | 1 | 1 | 0 | 1 | + | 77.7% |

Scoring: (+) Low Risk, (-) Moderate Risk (X) High Risk

| **Mixed Methods Appraisal Tool (MMAT), version 2018** | | |
| --- | --- | --- |
|  |  | **Citation** |
|  |  | (Ramaiya et al., 2022) |
|  | S1. Are there clear research questions? | 1 |
|  | S2. Do the collected data allow to address the research questions? | 1 |
| **1. Qualitative** | 1.1. Is the qualitative approach appropriate to answer the research question? | 1 |
|  | 1.2. Are the qualitative data collection methods adequate to address the research question? | 1 |
|  | 1.3. Are the findings adequately derived from the data? | 1 |
|  | 1.4. Is the interpretation of results sufficiently substantiated by data? | 1 |
|  | 1.5. Is there coherence between qualitative data sources, collection, analysis and interpretation? | 1 |
| **3. Quantitative nonrandomized** | 3.1. Are the participants representative of the target population? | 1 |
|  | 3.2. Are measurements appropriate regarding both the outcome and intervention (or exposure)? | 1 |
|  | 3.3. Are there complete outcome data? | 1 |
|  | 3.4. Are the confounders accounted for in the design and analysis? | 0 |
|  | 3.5. During the study period, is the intervention administered (or exposure occurred) as intended? | 1 |
| **Mixed methods** | 5.1. Is there an adequate rationale for using a mixed methods design to address the research question? | 1 |
|  | 5.2. Are the different components of the study effectively integrated to answer the research question? | 1 |
|  | 5.3. Are the outputs of the integration of qualitative and quantitative components adequately interpreted? | 1 |
|  | 5.4. Are divergences and inconsistencies between quantitative and qualitative results adequately addressed? | 0 |
|  | 5.5. Do the different components of the study adhere to the quality criteria of each tradition of the methods involved? | 1 |
|  | Overall | + |
|  | Weight (%) | 88.2% |

Scoring: (+) Low Risk, (-) Moderate Risk (X) High Risk

**Supplementary Table 3**

Study characteristics of the articles included in the systematic review

| **Citation** | **Total Sample**  **(Male/**  **Female)** | **Geographical Location** | **Regulation Measurement** | **Study design** | **Favouring Factors** | **Hindering Factors** |
| --- | --- | --- | --- | --- | --- | --- |
| Batool & Shafiq, 2022 [42] | 608  (Males- 330 Females=278) | Gujrat, Pakistan | SRQ, APRI, RSQ, & PYDI | Quantitative- Cross sectional study | Positive Peer Relationships  Effective Self-Regulatory Strategies | Bullying and Victimization  Rejection Sensitivity  Negative thinking |
| Ramaiya et al., 2022 [40] | Qualitative: 10 students, Quantitative: Intervention group (n=40), Control group (n=62) | Nepal | DERS, DBT-WCCL, BAI, CPSS, CFIS, Wagnild & Young Resilience Scale and Suicidal Ideation scale | A mixed-method, gender-stratified, non-randomized controlled design | Emotional regulation  Resilience  Coping skills | Anxiety,  Posttraumatic stress  Functional impairment |
| Uddin & Rahman, 2022 [43] | 250  (48% boys and 52% girls) | Dhaka, Bangladesh | CYVIC, ERQ, CYBA, & PIF | Quantitative-Cross sectional study | Cognitive Reappraisal  Expressive Suppression | Cyber victimization  Cyber aggression |
| Iqbal et al., 2023 [44] | 300  (Men-166, Women=134) | Lahore, Pakistan | Mother Overprotection Subscale of the EMBU-A scale, EDE-Q, Emotion Regulation Scale | Quantitative- Correlational research design | Not Reported | Mother Overprotection  Negative self-image  Emotional dysregulation |
| Akhtar & Bano, 2023 [45] | 500 adolescents  500 mothers | Gujrat, Pakistan | The social anxiety scale and Social-emotional competence scale | Quantitative- Cross sectional study | Positive maternal expressed emotions  Supportive maternal behaviors | Critical comments and hostility from mothers.  Negative maternal emotional expressions |
| Gunawardena & Schuck, 2021 [46] | 233 adolescents (Male-62 and Female-38)  349 parents | Colombo, Sri Lanka | DERS, Inventory of Parent-  Peer-Attachment-Parent Scale only (IPPA-R-Parent subscale-Sinhala version) and ADOTS-R | Quantitative-Cross sectional study | Strong parent-child attachment quality. | Parental ER difficulties  Lower Socio-Economic Status (SES) |
| P. Singh & Singh, 2013 [47] | 100  (Male-50 and Female-50) | India | DERS and AMS. | Quantitative- Cross sectional study. | Academic motivation and performance. | Difficulties in engaging in goal-directed activity.  Lack of emotional awareness.  Impulse control difficulties  Limited access to emotion regulation strategies.  Poor academic performance |
| Tanveer et al., 2023 [48] | 400  (50% boys and 50% girls) | 10-kilometer radius of Line Control (LOC), Sialkot Pakistan | DERS, Coping Strategies Scale for Adolescents, and Post-traumatic Stress Symptoms Checklist. | Quantitative- Cross sectional study. | Problem-focused coping strategies.  Spirituality as a coping mechanism | Emotion-focused coping.  Exposure to traumatic events |
| R. Singh et al., 2020 [49] | 384  (65.4% boys) | Birgunj, Nepal | SAS-A, DERS-18, and FFMQ | Quantitative- Cross sectional study. | Higher level of social support from close friends | Non-acceptance of emotional responses.  Lack of clarity regarding emotions.  Lack of emotional awareness. |
| Natso et al., 2018 [50] | 453  (Males-210 and Females-243) | India | STAXI-2CA, Rating scale on factors influencing anger, and BARQ-C | Quantitative- Correlational survey design | Use of coping strategies to manage anger effectively.  Social Impact  Emotional Self-Regulation  Gratification of Needs | Perception  Influence of Family Environment  Over-Expectations  Feelings of Frustration |
| Raval et al., 2018 [24] | 450  (45.5% females) | Gujrat, India | PAQ-R, Parent Response to Children’s Emotion Questionnaire, Children’s Sadness and Anger Management Scales-Adapted, Behavioural school engagement, | Quantitative- Cross sectional study | Adolescent School Engagement | Non-supportive parenting behaviors  Authoritarian Parenting |
| Janjhua et al., 2020  [51] | 110  (Male-66.36% and Female-33.64%) | Himachal Pradesh, India | Emotion Regulation Scale, Rosenberg Self Esteem Scale, and Feeling State Assessment | Quantitative- Cross sectional study | Yoga Practice | Not Reported |
| Kaul et al., 2019 [52] | 52  (Male-21 and Female-31)  52 Parents | Delhi, India | ERQ-CA, CCNES, SATI | Quantitative method | Supportive Parental Responses  Parental emotion socialization | Punitive Parental Responses |
| Saleem & Gul, 2018 [53] | 400  (Males-200 and Females-200) | Islamabad, Pakistan | ERQ | Quantitative- Cross-sectional study | Joint family systems: | Nuclear family systems |
| Zafar et al., 2021 [41] | 1500  (50% Female) | Punjab, Pakistan | DERS, PROMIS & BPFSC-11 | Quantitative- Cross-sectional study | Not Reported | Lack of Emotional Clarity  Impulse Control Difficulties  Non-Acceptance of Emotional Responses  Limited Access to Effective Emotion Regulation Strategies |
| Borthakur, 2019 [54] | 400  (Male-59.5%and Females-40.5%) | India | DERS-SF, IPR, & RAS | Quantitative- Correlation research design and Path Analysis | Supportive environments  Positively influence peer relationships | Negative peer interactions |
| G.B.Chaudhari, 2021 [55] | 100  (Male-50 and Female-50) | India | Emotional competence scale | Quantitative- Single group Pre-test and post-test design | Life skill | Not Reported |
| P. Singh & Singh, 2022 [56] | 617  (Male-356 and Female-261) | India | Youth Risk Behaviour Survey Questionnaire, Difficulties in emotion regulation questionnaire | 617  (Male-356 and Female-261) | Not Reported | Increased health-risk behaviors (unhealthy dietary habits, inadequate physical activity, self-harming behaviors, and risky driving) |
| Dash & Verma, 2017 [25] | 430  (Male-70% and Female-30%) | India | PAQ, ERQ, ECR-RS, & Intergenerational support scale | 430  (Male-70% and Female-30%) | Cognitive Reappraisal  Intergenerational Support | Authoritarian Parenting (attachment-related anxiety and avoidance)  Expression Suppression |
| Agarwal et al., 2023 [57] | 32 (Female) | Chandigarh, India | CERQ | Quantitative- Pre-post intervention study | Adaptive coping strategies | Maladaptive coping strategies |
| G. Singh, 2022) [58] | 120  (Males-60 and Females-60) | Chandigarh, India | NRIRQV, Inventory of parent and peer attachment revised and Emotional Regulation Questionnaire Satisfaction with life scale | Quantitative- Exploratory Cross-sectional design | Positive romantic relationship qualities  Cognitive Reappraisal | Expressive suppression  Negative romantic relationship qualities |
| Dubey et al., 2024 [26] | 740  (Males-185 and Females-185) | India | EIT and Criminal Propensity Scale. | Quantitative- Ex post facto research | Gender differences  Empathy  Handling relationships | Psychoticism  Criminal propensity |
| Sia & Aneesh, 2024 [59] | 385  (Males-179 and Females-206) | Kerala, India | Brief Resilience Scale  Psychological Well-being Scale  Multisource Assessment of Children’s Social Competence Scale & ERQ | Quantitative- Cross-sectional Study | Resilience  Cognitive Reappraisal  Prosocial Behavior | Expressive Suppression  Antisocial Behavior |
| P. Singh, 2024 [60] | 723  (Male-440 and Female-283) | Punjab, India | Youth Risk Behaviour Survey Questionnaire, DERS, Alabama Parenting Questionnaire | Quantitative- Cross-sectional Study | Perceived positive parenting practices | Perceived negative parenting practices  Health risk behaviors (HRBs) |

SRQ: Self-Regulation Questionnaire, APRI: Adolescent Peer Relations Instrument, RSQ: Rejection Sensitivity Questionnaire, PYDI: Positive Youth Development Inventory, DERS: The Difficulties in Emotion Regulation Scale, DBT-WCCL: Dialectical Behavior Therapy Ways of Coping Checklist, BAI: Beck Anxiety Inventory, CPSS: Child Posttraumatic Symptom Scale, CFIS: Child Functioning Impairment Scale, CYVIC: Cyber victimization questionnaire for adolescents, ERQ: The Emotion Regulation Questionnaire, CYBA: Cyber Aggression Questionnaire for Adolescents, PIF: Personal Information Form, EDE-Q: Eating Disorder Examination Questionnaire, ADOTS-R: Abbreviated version of the Dimensions of Temperament Survey-Revised, AMS- Academic Motivation Scale, SAS-A: Social Anxiety Scale for Adolescents, FFMQ: Five Factor Mindfulness Questionnaire, STAXI-2CA: State-Trait anger expression inventory, BARQ-C: Behavioral anger response questionnaire, PAQ-R: Parental Authority Questionnaire-Revised, ERQ-CA: Emotion Regulation Questionnaire for Children and Adolescents, CCNES: Coping with Children’s Negative Emotions Scale, SATI: School Age Temperament Inventory, PROMIS: Patient-Reported Outcomes Measurement Information System, BPFSC-11: Borderline Personality Features for Children–11-item scale, DERS-SF: Difficulties in emotional regulation scale- short form, IPR: Index of Peer Relation, RAS: Rathus Assertiveness Schedule, PAQ: Parental Authority Questionnaire, ECR-RS: Experience in close relationship structure questionnaire, CERQ: Cognitive Emotion Regulation Questionnaire, NRIRQV: Network of Relationships Inventory-Relationship Qualities Version, EIT: The Emotional Intelligence Test.
